# Evaluation of Safety and Beneficial Health Effects of the Human-Milk Strain *Bifidobacterium breve* DSM32583: An Infant Pilot Trial

**DOI:** 10.3390/nu16081134

**Published:** 2024-04-11

**Authors:** Claudio Alba, Marta Carrera, Guillermo Álvarez-Calatayud, Rebeca Arroyo, Leónides Fernández, Juan M. Rodríguez

**Affiliations:** 1 Department of Nutrition and Food Science, Complutense University of Madrid, 28040 Madrid, Spain; c.alba@ucm.es (C.A.); rebecaa@vet.ucm.es (R.A.); 2 Centro de Atención Primaria Silvano, Comunidad de Madrid, 28043 Madrid, Spain; marta.carrera@salud.madrid.org; 3 Department of Pediatric Gastroenterology, Hospital Universitario Gregorio Marañón, 28007 Madrid, Spain; galvarezcalatayud@gmail.com; 4 Department of Galenic Pharmacy and Food Technology, Complutense University of Madrid, 28040 Madrid, Spain; leonides@ucm.es

**Keywords:** *Bifidobacterium breve*, probiotics, infant formula, safety, bifidobacteria, short-chain fatty acids, infections

## Abstract

Human milk promotes the growth of bifidobacteria in the infant gut. Adding bifidobacterial species to infant formula may contribute to increasing their presence in the gut of formula-fed infants. Therefore, the safety and anti-infectious effects of *Bifidobacterium breve* DSM32583, a breast milk isolate, were assessed in a pilot trial involving 3-month-old infants. The infants were randomly assigned to either the probiotic (PG) or the control (CG) groups. All the infants consumed the same formula, although it was supplemented with the strain (1 × 10^7^ cfu/g of formula) in the PG. Overall, 160 infants (80 per group) finished the intervention. Infants in CG gained more weight compared to PG (*p* < 0.05), but the weights for age Z-scores at 6 months were within the normal distribution for this age group. The rates of infections affecting the gastrointestinal and respiratory tracts and antibiotic therapy were significantly lower in the PG. The bifidobacterial population and the level of short-chain fatty acids were higher (*p* < 0.05) in the fecal samples of PG infants. No adverse events related to formula consumption were observed. In conclusion, the administration of an infant formula with *B. breve* DSM32583 was safe and exerted potential beneficial effects on gut health.

## 1. Introduction

Gut colonization in early life exerts a lifelong influence on the host’s health [1]. Breastfeeding is considered a key factor for the proper establishment of the gut microbiota since human milk contains several interactive compounds and cells with the ability to drive and modulate this process [2,3,4,5,6]. Among such compounds, human-milk oligosaccharides (HMOs) seem particularly relevant since they are responsible for the typical predominance of bifidobacteria in the guts of breast-fed babies [7,8,9,10]; in fact, these compounds were historically known as the “bifidogenic factors” until their chemical composition was unveiled [11,12]. The analysis of the genome of several bifidobacterial species and strains isolated from infant feces has revealed that these bacteria are genetically adapted to use HMOs, being an excellent example of host–microbe coevolution and natural selection [13,14,15]. In addition, breast milk may carry live bifidobacteria [16,17,18,19], and, therefore, this biological fluid can be considered a complex symbiotic food.

A variety of perinatal factors may affect the development of the gut microbiome. Cesarean section has been frequently associated with a gut dysbiosis state in the infant gut, characterized by a depletion in bifidobacterial populations [20,21,22], and a higher incidence of infections [23], when compared with vaginally-delivered infants. In these cases, the bifidobacterial populations can be restored through breastfeeding, a practice that leads to a decrease in the infection rate [23]. In contrast to human milk, traditional formulae have a negative impact on the composition of the gut microbiota early in life, which is usually characterized by a depletion in the population of such bifidobacterial species [24]. As a consequence of these and other factors, bifidobacteria may be not detected or at very low levels in the feces of some infants [25,26,27,28]. The will to reduce this gap has prompted the development of new formulas by incorporating bioactive ingredients, such as probiotics, prebiotics, or postbiotics [29,30]. Since their introduction to the market in the early 1980s, bifidobacteria-containing products have been widely commercialized as probiotic supplements for the infant population, including several *B. breve* strains [31,32]. In previous trials, the use of *B. breve* strains (alone or combined with prebiotic substances and/or with strains belonging to other bacterial species) to infants, including healthy ones but, also, infants with a variety of conditions (prematurity, allergic disorders, celiac disease chemotherapy, and antibiotic-associated diarrhea) has been shown to be safe, well-tolerated, and has led to beneficial health outcomes [33,34,35,36,37,38,39,40,41,42,43,44,45].

The development of infant formula that contains bifidobacterial strains isolated from human milk seems particularly appealing, and, in this context, the objective of this work was to test the safety and efficacy of *B. breve* DSM32583 for preventing gastrointestinal and respiratory tract infections in a population of 3-months-old formula-fed infants.

## 2. Materials and Methods

### 2.1. Study Design and Population

This trial was designed as a randomized double-blind controlled intervention with two groups. Healthy, full-term, three-month-old infants, who had been exclusively formula-fed from birth, were recruited into the study. Two centers (Primary Care Center Silvano and Hospital Gregorio Marañón; Madrid, Spain) participated in the design and recruitment, which started in March 2010 and finished in September 2011. The mothers of the recruited infants had voluntarily chosen to formula-feed their infants from birth despite repeated advice and encouragement to breastfeed their babies due to the associated benefits this feeding option provides for both the infant and the mother. Hospital Gregorio Marañón is accredited by the WHO and UNICEF for its work promoting breastfeeding.

Exclusion criteria included a history of mild or serious gastrointestinal disorders (history of chronic diarrhea or constipation, gastroesophageal reflux), gastrointestinal surgery, cow’s milk protein allergy, metabolic disorders (diabetes, lactose intolerance), immune deficiency, antibiotic prescription three weeks prior to inclusion and previous use of probiotic-containing formula. Exclusion criteria during the study were lack of compliance with the study protocol, adverse events related to the consumption of the study formula, not attending scheduled visits to the primary care center, and severe regurgitation and/or colic that, according to pediatricians, needed prescription of a special formula. Complementary foods were not introduced until the end of the participation in the trial when all recruited infants were <26 weeks old. This practice fits with ESPGHAN recommendations [46].

The Ethics Committee of the Hospital Clínico (Madrid, Spain) approved the protocol (reference 10/017-E). Parents or caregivers provided written informed consent before starting the intervention. The trial was retrospectively registered in the US Clinical Trial Database (www.clinicaltrial.gov) (NCT05973812).

### 2.2. Sample Size and Randomization

The sample size was estimated based on the primary outcome of average weight gain of infants between baseline visit (90 ± 5 days of age) and visit 1 (180 ± 5 days of age). The infants’ weight gain was estimated to be ~16.7 g per day with an SD of ~3.5 g. The trial was designed with the power to detect differences in weight gain equal to 0.5 standard deviations on the basis of previous publications [43,44]. For this purpose, each group should include ≥65 infants under the assumption of non-inferiority (one-sided test; significance level: 2.5%; power: 80%).

The recruited infants were randomly assigned into one of the two study groups (probiotic group [PG] and control group [CG]), using a computer program (SIGESMU^®^). All study personnel (except for the statistician of the research group) and parents remained blinded during the whole trial.

### 2.3. Study Formula

A total of 187 infants were randomized into one of the two groups (probiotic group [PG] and control group [CG]). CG infants received a commercial infant formula (HiPP Biológico, Leche de Inicio HiPP 1, 600 g) with a nutritional composition that fulfilled the EU regulations (EU RL 2006/141) current at the time of study implementation. PG infants received the same formula but supplemented with *B. breve* DSM32583 at a concentration dose of 1 × 10^7^ cfu/g of powdered formula. The strain was provided by Biopolis (Valencia, Spain). Both formulae were the only food ingested by the respective infants for 3 months. The standard commercial HiPP infant formula was purchased and transferred to plain white undistinguishable tins, and, then, those aimed for the PG infants were supplemented with the strain DSM32583. Each formula was prepared in a clean room in the facilities of the Dpt. of Galenic Pharmacy and Food Technology at the Complutense University of Madrid. The tins were provided with a neutral label on which only the randomization number and the expiry date were printed. In addition, parents received the feeding instructions that accompanied the original food cans. These explained the preparation and feeding method but contained no reference to the substances studied in the trial. This ensured that both the parents and the study teams were blinded to the Investigational Product.

Ten tins of each group were submitted to two microbiological quality controls. The first after preparation (and before giving the test products to the parents) and the second after storage for 3 months at room temperature. For this purpose, 25 g of the powder were diluted in sterile peptone water (1:10 *w*/*v*). After homogenization, proper dilutions were spread onto plates of the following media: MacConkey (MCK, BioMérieux, Marcy l’Etoile, France, isolation of enterobacteria), Columbia Nadilixic Acid (CNA, BioMérieux, streptococci, enterococci, staphylococci, and related Gram-positive bacteria), Sabouraud Dextrose Chloramphenicol (SDC, BioMérieux; yeasts), Polymyxin Pyruvate Egg yolk Mannitol Bromothymolblue Agar (PEMBA, BioMérieux; *Bacillus*), and De Man, Rogosa and Sharpe (MRS, Oxoid) supplied with 0.05% (*w*/*v*) L-cysteine (MRS-Cys; isolation of lactic acid bacteria and bifidobacteria). CNA, MCK, SDC, and PEMBA plates were incubated aerobically at 37 °C for 48 h while MRS-Cys plates were incubated in anaerobiosis (85% nitrogen, 10% hydrogen, 5% carbon dioxide) at 37 °C for 48 h. Then, the colonies in each medium were enumerated, and at least one representative of each morphology was identified by MALDI-TOF mass spectrometry.

### 2.4. Study Outcomes

The infants were evaluated immediately before starting the assay and immediately after the end of the intervention. The primary outcome was average weight gain during the trial. Average length and head circumference gain, incidence of intestinal and respiratory infections, fecal concentration of bifidobacteria, and fecal levels of short-chain fatty acids (SCFA) were included as secondary outcomes. Gastrointestinal infection (GI infection) was defined as loose or watery stools at least three times per day with or without fever or vomiting [47] while respiratory tract infections were defined as the presence of abundant mucosity and/or cough during two or more consecutive days with or without fever or the presence of wheezing and/or crepitants with or without fever [48]. Otitis cases were included within respiratory tract infections because of the involvement of otopathogens that are typically associated with the upper respiratory tract (*Streptococcus pyogenes*, *Streptococcus pneumoniae*, *Haemophilus influenza*, *Moraxella catarrhalis*, and others) in both acute otitis media (AOM) and recurrent acute otitis media (rAOM) [49,50]. The medical dictionary for regulatory activities (MedDRA) was used to code for adverse events (AEs).

### 2.5. Collection and Analysis of Fecal Samples

Fecal samples were obtained by staff of the study centers at both study visits. Each fecal sample was aliquoted in three parts and stored at −20 °C until analysis. Samples were transported to the research laboratory by a courier service within containers containing dry ice. Once in the lab, two aliquots were used for bifidobacterial and short-chain fatty acid quantification, respectively, and the third aliquot was preserved at −80 °C.

The quantification of the bifidobacteria present in the fecal samples was performed on MRS-Cys agar plates, as described above. Bifidobacterial enumeration was restricted to Gram-positive and catalase-negative colonies that cellular extracts showed fructose-6-phosphate phosphoketolase (F6PPK) activity [51]. PCR was used to detect the presence of *B. breve* DNA in the samples using a previously described method [52].

For quantification of short-chain fatty acids (SCFA), the samples were homogenized with 150 mM NaHCO_3_ (pH 7.8) (1:5, *w*/*v*) in an argon atmosphere. Samples were incubated for fermentation during 24 h at 37 °C and stored at −80 °C until the extraction. The analysis of SCFA was performed by gas chromatography as previously described [53].

### 2.6. Statistical Analysis

The quantitative assays included in this work were performed, at least, in triplicate. The modified intention-to-treat (mITT) analysis included randomized infants who were deemed eligible after randomization, consumed the study formula at least once, and provided at least one measurement for the primary outcome. The mITT data set was used for the analysis of the primary outcome and the secondary parameters.

R software (R Core Team) was employed for the statistical analyses. Anthropometric measurement data were expressed as the mean and standard deviation (SD), while fecal bacterial counts and SCFA values were expressed as the median and interquartile range (IQR). Anthropometric data were transformed to age- and gender-standardized Z-scores using the WHO-recommended methodology and the R package anthro version 1.0.0 [54]. The Student’s *t*-test was employed to compare means by group of z scores for weight, length, and head circumference at the 6-month visit. A linear mixed model using infant formula and sex as fixed factors and weight as the baseline as a covariate was applied to explore differences in total weight gain between the baseline and the end of the intervention.

Chi-square tests were applied to compare the incidences of gastrointestinal tract (GIT) infections, respiratory tract (RT) infections, and antibiotic usage between both trial groups, with exact *p*-values calculated using the Mid-p exact method due to sample size constraints. Additionally, odds ratios and relative risks were calculated, with the two-sided 5% significance level and 95% confidence intervals obtained for the estimates, which were computed via the Taylor series approximation. Incidence rates were further compared using Poisson regression to address the quantitative nature of the data. In exploring associations between the *Bifidobacterium* level and the incidence of GIT and RT infections, as well as antibiotic use, binary logistic regression models were developed. These models were constructed using the family = binomial parameter, where the *Bifidobacterium* count (log_10_ cfu/g) was the independent variable, and the binary health outcomes served as dependent variables. The Mann–Whitney U non-parametric test was used to compare fecal bacterial counts. The tests were performed at the two-sided 5% significance level, and 95% confidence intervals were obtained for the estimates. Differences were considered significant at *p* < 0.05.

## 3. Results

### 3.1. Study Population

**Figure 1:**
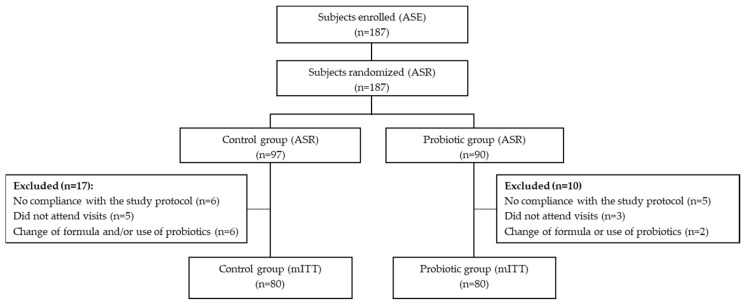
Flow chart of infants participating in the study. ASE, all subjects enrolled; ASR, all subjects randomized; mITT (modified intention-to-treat).

A flow chart including all the participants in this trial is shown in Figure 1. A total of 187 infants were enrolled in the study and randomized. Among them, 27 randomized subjects (10 PG, 17 CG) were excluded from the mITT population due to reasons specified in Figure 1. The baseline characteristics of the 160 infants (CG: 80: PG: 80) who fulfilled the whole intervention period and were therefore included in the mITT population were similar in both groups (Table 1).

### 3.2. Quality Control of the Infant Formula

Ten control and ten probiotic-supplemented tins were analyzed immediately after the addition of the probiotic and re-packaging, as well as after their storage for 3 months at room temperature. In the case of the control tins, no microbial growth was detected in any of the tested media. In relation to the DSM32583-containing tins, bacterial growth was only observed in the MRS-Cys plates for bifidobacterial quantification. All the isolates retrieved from these plates belonged to the *B. breve* species. The concentration ranged from 7.04 to 7.15 log_10_ cfu/g in the first sampling time and from 6.95 to 7.08 log_10_ cfu/g at the end of the storage period.

### 3.3. Anthropometric Measurements

Regarding the weight gain, infants in the CG group gained slightly more total weight over the 3-months intervention period and had slightly higher weight-for-age Z-scores in comparison to the PG group, but both groups showed weight-for-age Z-scores that were within the normal weight distribution for this age group. No significant differences were observed for head circumference (Table 2). No differences were detected between both groups in relation to the length of infants at 3 months of age; however, PG infants were significantly taller and had a higher length-for-age Z-score at 6 months of age than those in the CG group (*p* < 0.001). (Table 2).

### 3.4. Infection Parameters

A total of eight infants (six in the CG and two in the PG discontinued the intervention due to strong reflux/regurgitation/colic and had to change to an anti-reflux infant formula or receive a probiotic (Reuteri^®^).

The intervention led to differences (*p* < 0.05) in the incidence rates of both respiratory and GI infections between both groups (Table 3). In relation to those affecting the respiratory tract, the PG showed a significant (56.3%) reduction in the incidence rate (IR: 0.310) in comparison to the CG (IR: 0.710) (Table 3). Regarding GI infections, there was a significant reduction (73.9%) in the incidence rate (IR: 0.075) of PG compared to CG (IR: 0.287) (Table 3). The incidence-rate ratio (IRR) for both respiratory (IRR = 0.437) and GI infections (IRR = 0.261) was different (*p* < 0.05), revealing that in the CG the incidence rate of both types of infections was higher than in the PG. In addition, differences (*p* < 0.05) were also found in the use of antibiotics between both groups (PG: IR = 0.05; CG: IR = 0.175; IRR = 0.286), with an IR reduction of 71.4% in PG compared to CG. Most of the adverse effects reported during the trial were referred to the infections described above. The remaining adverse effects (<5%) were mainly the consequence of domestic accidents and were not related to the assayed product.

### 3.5. Bifidobacterial Count and SCFA Concentration

**Figure 2:**
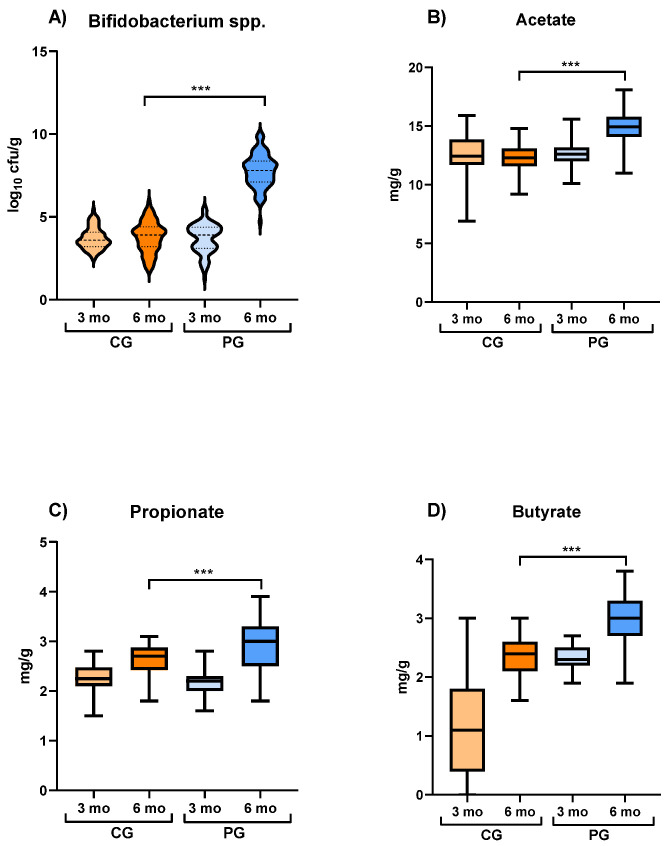
Median bifidobacterial counts in fecal samples of infants (as log_10_ cfu/g) (**A**), and fecal concentration of short-chain fatty acids (mg/g feces) (**B**–**D**) at 3 and 6 months of age. Mann–Whitney U was used to evaluate differences in median values between the control and probiotic groups at 6 months. Data expressed as median (IQR). ***, *p* < 0.05.

Total bifidobacterial counts were similar in both groups at baseline (CG: 3.57 ± 0.8 log_10_ cfu/g; PG: 3.94 ± 1.21 log_10_ cfu/g). The bifidobacterial concentration remained similar in CG at the end of the study (3.93 ± 1.21 log_10_ cfu/g) but, in contrast, it increased significantly in PG (7.84 ± 1.23 log_10_ cfu/g) and was different between both groups after the trial (*p* < 0.001) (Figure 2A). The number and percentage of samples from which *B. breve* could be detected were also similar in both groups at baseline (CG: 7 samples [9%]; PG: six samples [7%], *p* > 0.05). These values were similar in CG at the end of the intervention (nine samples [11%], *p* = 0.652) but, again, they increased significantly in PG (73 samples [91%], *p* > 0.001). Finally, the number and percentage of positive samples for *B. breve* species-specific PCR were slightly higher than those obtained by culture-based methods (CG: 12 samples [15%] at baseline and 15 samples [17%] at the end of the study, *p* = 0.415; PG: 10 samples [12%] at baseline and 75 samples [94%] after the end of the trial, *p* < 0.001).

The potential impact of the *Bifidobacterium* load on GI and respiratory tract infections, and on antibiotic use was assessed using a logistic regression model. Our results indicate that an increase in the *Bifidobacterium* levels was linked with a significant decrease in both GI and respiratory tract infections (GI infections: −1.2608, *p* < 0.001; respiratory infections: −0.5833, *p* < 0.001). In the case of antibiotic use, the negative estimate of −0.3735 with a *p*-value of 0.0231 implies a less pronounced yet still significant association between an increase in the *Bifidobacterium* concentration and a reduction in antibiotic use (Table 4).

The fecal concentration of SCFA (butyrate, propionate, and acetate) was similar in both groups at baseline (acetate: CG: 12.48 ± 2.13 mg/g, PG: 12.58 ± 1.09 mg/g; propionate: CG: 2.26 ± 0.3 mg/g, PG: 2.21 ± 0.34 mg/g; butyrate: CG: 1.12 ± 1.36 mg/g, PG: 2.31 ± 0.3 mg/g), but were higher in the PG than in the CG at the end of the probiotic intervention (acetate: CG: 12.27 ± 1.5 mg/g, PG: 14.96 ± 1.68 mg/g; propionate: CG: 2.66 ± 0.38 mg/g, PG: 2.98 ± 0.77 mg/g; butyrate: CG: 2.38 ± 0.47 mg/g, PG: 3.0 ± 0.57 mg/g) (Figure 2B–D).

## 4. Discussion

Bifidobacteria dominate the gastrointestinal microbiota during infancy and are associated with lifelong health benefits. Therefore, providing bifidobacterial strains to infants who may be deprived of these strains is an important approach. Here, a pilot trial for the assessment of the safety and efficacy of *B. breve* DSM32583 in 3-month-old infants was conducted.

In line with the literature, the results of this 3-month intervention demonstrated that consumption of the probiotic *B. breve* DSM32583 was safe since no severe adverse events were detected. Although total weight gain after the intervention was lower in the probiotic group, all infants grew according to WHO standards, as evidenced by Z-scores falling within the normal weight distribution for this age group. Although the results of previous studies indicate that ingestion of probiotics during infancy is likely to either maintain or increase weight gain when compared to a placebo [48,55], other works found that some probiotic strains may have the opposite effect and, therefore, may be useful to inhibit excessive weight gain during the first years of life [56]. In fact, higher weight gain is not necessarily a health benefit since breastfed infants (i.e., the gold standard) usually have a lower weight gain in comparison to formula-fed infants in the first months of life. Since the weight-for-age Z-scores fall within the normal ranges for infants in this age range and total weight gain corresponds to the proposed weight gain of 400 to 560 g per month between 3 and 6 months of age (PG group 473 g per month), the weight gain observed on the PG group can be considered normal weight gain for infants.

Oral intake of *B. breve* DSM32583 for 3 months increased the bifidobacteria and SCFA concentrations in the fecal samples. A previous study showed that the administration of probiotic bacteria, including bifidobacteria, to preterm neonates decreased the abundance of *Escherichia*, *Klebsiella*, and *Enterococcus*, and increased that of *Bifidobacterium*, reducing the incidence of necrotizing enterocolitis (NEC) [57].

The increase in the production of SCFAs may be of relevance, especially regarding the improvement of gastrointestinal health. SCFAs play key roles in the gut, from being the primary source of energy to colonocytes and participating in water and mineral absorption, to contributing to enhancement of the barrier function through increasing mucin biosynthesis, immunomodulation, and protection against pathogens [58,59]. Therefore, SCFAs can directly impact host health, and, in fact, increasing SCFA levels in the gut is one of the main mechanisms enabling probiotic strains to exert positive effects on host health [60].

In this work, a logistic regression model indicated that the increase in the *Bifidobacterium* levels was associated with a significant reduction in both GI and RT infections. It has been repeatedly reported that gut bifidobacteria participate in the barrier effect against enteric pathogens through different mechanisms, including antimicrobial activity, enhancement of immune responses, and/or regulation of the signaling pathways involved in the integrity of the tight-junction barrier [61,62,63,64,65]. It has been reported that two *B. breve* strains alleviated DSS-induced colitis in a murine model by maintaining the intestinal epithelial barrier, inhibiting the inflammatory cytokines, and modifying the composition of the gut microbiome [66]. It was also found that *B. breve* UCC2003 may play a pivotal role in the correct development of the intestinal epithelium during early life by extensive regulation of the intestinal epithelial cells (IECs) transcriptome [67]. 

Regarding RT infections, a systematic review concluded that a low relative abundance of gut bifidobacteria in the first 12 months of life was associated with childhood respiratory diseases, including respiratory infections [68]. Previously, another *B. breve* strain affected respiratory disease susceptibility in a mouse model of asthma and respiratory infection [69]. Another study described that those mice with a higher bifidobacterial abundance in their feces displayed a higher survival rate when challenged with the influenza virus, probably because of the prevention of an excessive neutrophil influx to the airways [70].

The infant trial has both strengths and limitations. Both groups had the same number of infants in the mITT, and the fact that none of the mITT infants received complimentary feeding before the end of the study may also be considered a strength since the introduction of other foods may have an impact on the composition of the infant gut microbiota and the fecal SCFA concentration. In addition, it was confirmed that the concentration of *B. breve* DSM32583 remained stable for the 3-month intervention period.

However, the trial also faces some limitations. Data about the potential changes in the main physical characteristics of infant stools are lacking, and no information on tolerance of or parental satisfaction with the study formula or amount of formula intake is available. Moreover, infants were recruited at 3 months of age, when they had already been consuming an infant formula prior to the intervention. Other studies that focused on the effects of adding probiotic strains to infant formulae started the intervention earlier in life (from immediately after birth to 1-month-old infants) to avoid overlapping with the period of introduction of complementary foods [71,72] and to assess the addition of the *B. breve* strain to infant formula in young infants right after birth. The results of our data in this study, therefore, cannot be generalized for healthy young infants 0–3 months of age. Although, as stated above, complimentary food was not started before the end of the intervention, it is possible that an earlier intervention may have a strong impact on gut colonization.

Finally, this study was conducted in 2010–2011, according to the current scientific standard of data collection for nutritional studies and sample analysis at that time. The main reason why it has taken so long from the end of the trial to the publication is that, shortly after this pilot trial was finished, the probiotic strain was acquired by a company to be further developed and added to infant formula and, due to internal company strategies, the publication of any data relating to the strain was delayed. We are aware that microbiome research has evolved enormously since then, moving from describing microorganisms belonging to the human microbiota to elucidating their functional roles and their highly personalized interactions with the host [73]. Technical improvements, from sequencing technologies, and culturomics to transcriptomics, metabolomics, and bioinformatics, have revolutionized the workflow of microbiome research, facilitating extensive study of the functions and mechanisms of the microbiome [74]. Such approaches can be applied nowadays to samples obtained in clinical trials, enabling a better knowledge about the mechanisms of action of a given strain and why the health outcomes may be different depending on the host, opening the possibility of future personalized interventions [75].

This trial was conducted to investigate the safety and health benefits of a single strain (mono-strain formulation). We are also aware that there is a growing trend to use a combination of two or more probiotic strains in commercial infant formulas (multi-strain formulations) [76] and/or to add prebiotics (synbiotic formulations). Multi-strain formulations are based on the synergistic or complementary interactions that may be established between different strains, enhancing the possibility of beneficial outcomes for a host. The addition of prebiotic substances may be directed to support specific members of the autochthonous microbiota of the host (complementary synbiotic) or to promote the growth, persistence, or activities of the specific strain(s) included in the formula (complementary synbiotic) [77]. Some bifidobacterial strains, either alone or together with other strains or prebiotics, have shown health benefits for the infant population [78]. However, only a few studies have compared the effect of a multi-strain probiotic or a synbiotic with that of the different strains or components individually [79]. In some cases, the multi-strain probiotic or the synbiotic product was more effective than the respective mono-strain probiotics [80], while other in vivo studies have provided conflicting results [79]. It must be considered that some strains are incompatible with the same product [79] and that the combination of multiple strains and prebiotics represents a difficult biotechnological challenge for formulation design, industrial production, stability, traceability, quality control, and recognition of the safety issues and benefits provided by each component of the resulting product [81,82,83]. Anyway, the safety and efficacy of a multi-strain probiotic or a symbiotic product should ideally be tested for each individual component and their final combination, and, in this frame, our study provides the first pilot data that *B. breve* DSM32583 represents a safe candidate for the addition to infant formula. However, work is in progress to elucidate if its effects may be enhanced by combining it with prebiotics and/or other strains.

## 5. Conclusions

In conclusion, *B. breve* DSM32583 points towards the potential beneficial health outcomes observed in the pilot trial included in this work. This work can be considered as a “proof of concept” for the safety of the infant population. These findings, as well as the potential health benefits, must be confirmed and explored in future studies, including trials with a longer intervention period, younger infants, and infants and children suffering from conditions that are characterized by disturbances in the gut microbiota.

## Figures and Tables

**Table 1:** Baseline characteristics of the infants that completed the study.

Characteristic	CG (*n* = 80)	PG (*n* = 80)
Male/female, *n* (%)	47/33 (59/41)	44/36 (55/45)
Age at enrolment (weeks), mean ± SD	12.1 ± 0.6	12.1 ± 0.7
Birth weight (kg), mean ± SD	3.29 ± 0.34	3.27 ± 0.40
Delivery by C-section *n* (%)	21 (26)	20 (25)
Gestational age (weeks) mean ± SD	40.3 ± 1.4	40.5 ± 1.3
Age of mother at birth (years) mean ±SD	28.9 ± 5.1	29.7 ± 5.5
No breast feeding ^a^, *n* (%)	80 (100%)	80 (100%)
Older siblings, *n* (%)	41 (52)	47 (49)
Weight of mother (kg) mean ± SD	69.7 ± 6.2	68.9 ± 5.8

^a^ no breast feeding refers to no single breast feeding meal since birth.

**Table 2:** Anthropometric measurements at baseline (3 months) and at the end of the intervention (6 months of age) for the mITT population. Values presented as means ± SD.

Growth Parameters	Control Group (*n* = 80)				Probiotic Group (*n* = 80)				*p*-Value
	3 Months		6 Months		3 Months		6 Months		
	Values	Z-score	Values	Z-score	Values	Z-score	Values	Z-score	
Weight (kg)	6.4 ± 0.7	0.38 ± 0.77	8.1 ± 1.1	0.73 ± 1.28	6.5 ± 0.8	0.83 ± 0.73	8.0 ± 1.0	0.32 ± 1.2	0.04 ^1^
Total weight gain	1.82 ± 0.13				1.42 ± 0.13				0.03 ^2^
Length (cm)	62.2 ± 3.6	0.92 ± 1.57	67.3 ± 3.2	0.33 ± 0.53	61.5 ± 1.6	0.8 ± 0.89	69.7 ± 2.9	1.67 ± 1.45	<0.001 ^1^
Head circumference (cm)	39.6 ± 2.9	−0.28 ± 2.46	43.8 ± 1.4	0.8 ± 1.19	39.4 ± 3.3	−0.58 ±2.77	44.1 ± 1.5	1.16 ± 1.37	0.08 ^1^

^1^ *t*-test to evaluate differences in Z-scores at 6 months comparing CG and probiotic group PG. ^2^ Linear mixed models to evaluate the difference in total weight gain using baseline weight as a covariate.

**Table 3:** Comparative outcomes for gastrointestinal tract (GIT) infections, respiratory tract (RT) infections, and antibiotic use (AU) between the control (CG) and the probiotic (PG) groups.

		Control Group	Probiotic Group	*p*-Value	Relative Risk (CI 95%)	Odds Ratio (CI 95%)	Incidence Rate Ratio (IRR)	IR Decrease (%)
GIT infections	Infants (*n*)	14	4	0.013	0.29 (0.10–0.83)	0.25 (0.07–0.77)		
	Events (*n*)	23	6					
	Incidence rate (IR)	0.29	0.07				0.261	73.9
RT infections	Infants (*n*)	36	18	<0.001	0.50 (0.31–0.80)	0.36 (0.18–0.71)		
	Events (*n*)	57	25					
	Incidence rate (IR)	0.71	0.31				0.437	56.3
Antibiotic use	Infants (*n*)	8	4	0.049	0.50 (0.16–1.59)	0.48 (0.12–1.64)		
	Events (*n*)	14	4					
	Incidence rate (IR)	0.17	0.05				0.286	71.4

**Table 4:** Potential impact of the *Bifidobacterium* concentration on GI and respiratory tract infections, and on antibiotic use as assessed using a logistic regression model. AIC, Akaike information criterion.

	Intercept Estimate	Pr(>|z|) Intercept	*Bifidobacterium* Estimate	Pr(>|z|) *Bifidobacterium*	AIC
Respiratory infections	2.4378	7.98 × 10^−6^	−0.5833	1.83 × 10^−8^	165.08
GI infections	3.1864	0.00452	−1.2608	8.12 × 10^−5^	76.827
Antibiotic treatments	−0.6265	0.4245	−0.3735	0.0231	82.986

## Data Availability

The data presented in this study are available upon reasonable request from the corresponding author. The data are not publicly available due to the will to know the interest/field of the researchers and clinicians who request the data.
